# Supplementary material for: Cistracurium Besylate 10 mg/mL Solution Compounded in a Hospital Pharmacy to Prevent Drug Shortages: A Stability Study Involving Four Degradation Products
Source: Pharmaceutics. 2023 May 4;15(5):1404. doi: 10.3390/pharmaceutics15051404 (PMC10221331; doi:10.3390/pharmaceutics15051404)
Supplement: Supplementary file 1 [file pharmaceutics-15-01404-s001.zip › pharmaceutics-2340920-supplementary.pdf]

Cistracurium besylate 10 mg/mL solution compounded in a hospital pharmacy to prevent drug shortages: a stability study involving four degradation products

Supplementary data – table of contents and captions

S1 **Figure S1**. Chromatograms after degradation of cisatracurium by 0.005N NaOH. (λ = 280nm). T0: immediately after the addition of NaOH; T10: After 10 minutes of exposure.

S2 **Figure S2**. Chromatograms after degradation of cisatracurium by 1N HCl. (λ = 280nm). T0: immediately after the addition of HCl; T3h: after 3 hours of exposure.

S3 **Figure S3**. Chromatograms after the heat degradation of cisatracurium. (λ = 280nm). T0: immediately after the start of heat exposure, T2h: After 2 hours of exposure at 105°C, T3h: After 3 hours of exposure at 60°C.

S4 **Figure S4**. Chromatograms after degradation of cisatracurium by 30% H2O2 at 60°C. (λ = 280nm). The arrows indicate the peak corresponding to H2O2. T0: immediately after the addition of H2O2; T2h: After 2 hours of exposure.

S5 **Figure S5**. Chromatograms after UV degradation of cisatracurium at 254 nm. (λ = 280nm). T0: immediately after the start of the UV exposure; 96h: after 96 hours of exposure.

S6 **Table S1**. Stability study values at each time point - Data are given as mean ± standard deviation.

S7 **Figure S6**. Linear fit in accordance with ICH Q1E.

S8 **Figure S7**. MS/MS spectrum of cisatracurium (m/z = 464 (M2+)), collision energy = 30 eV.

S9 **Figure S8**. MS/MS spectrum of DP1, EP impurity A (m/z= 430 (M+)), collision energy = 35 eV.

S10 **Figure S9**. MS/MS spectrum of laudanosine, EP impurity C (m/z= 358 (M+H+)), collision energy = 25 eV.

S11 **Figure S10.** MS/MS spectrum of DP2, EP impurity E and/or F (m/z= 516 (M+)), collision energy = 40 eV.

S12 **Figure S11**. MS/MS spectrum of DP3, EP impurity N and/or O (m/z= 570 (M+)), collision energy = 40 eV.

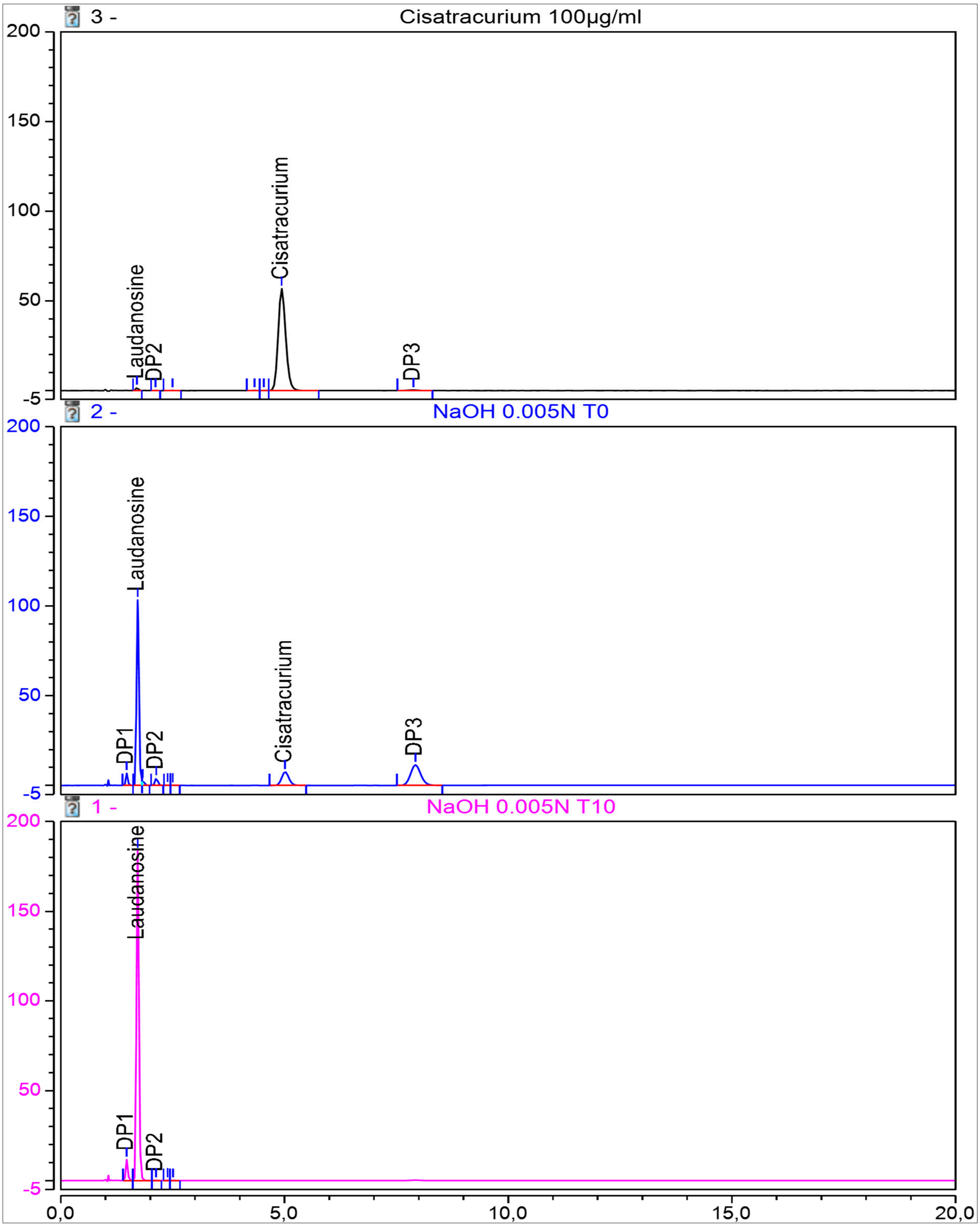

Figure S1. Chromatograms after degradation of cisatracurium by 0.005N NaOH. (λ = 280nm).

T0: immediately after the addition of NaOH; T10: After 10 minutes of exposure.

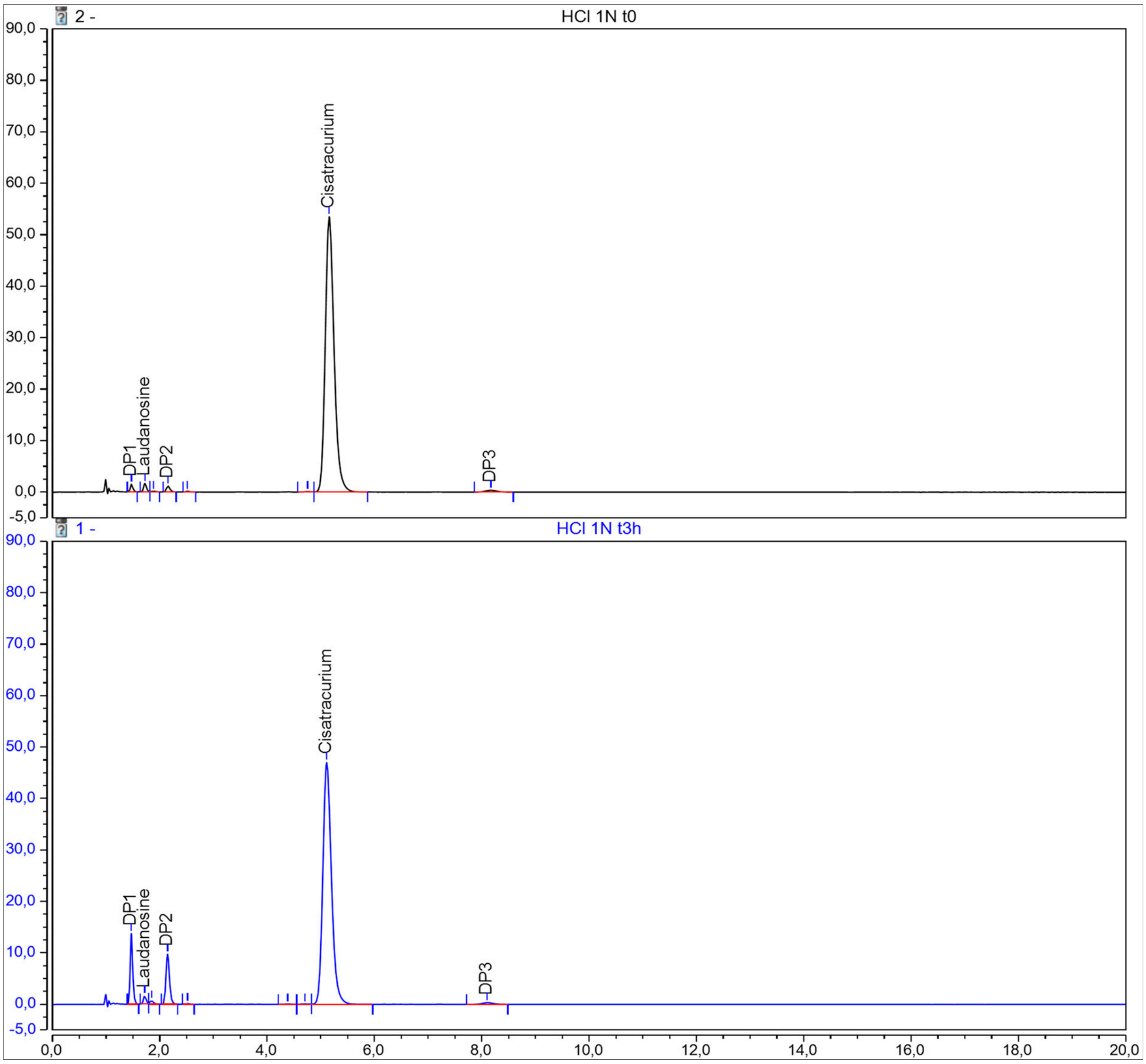

Figure S2. Chromatograms after degradation of cisatracurium by 1N HCl. (λ = 280nm). T0: immediately after the addition of HCl; T3h: after 3 hours of exposure.

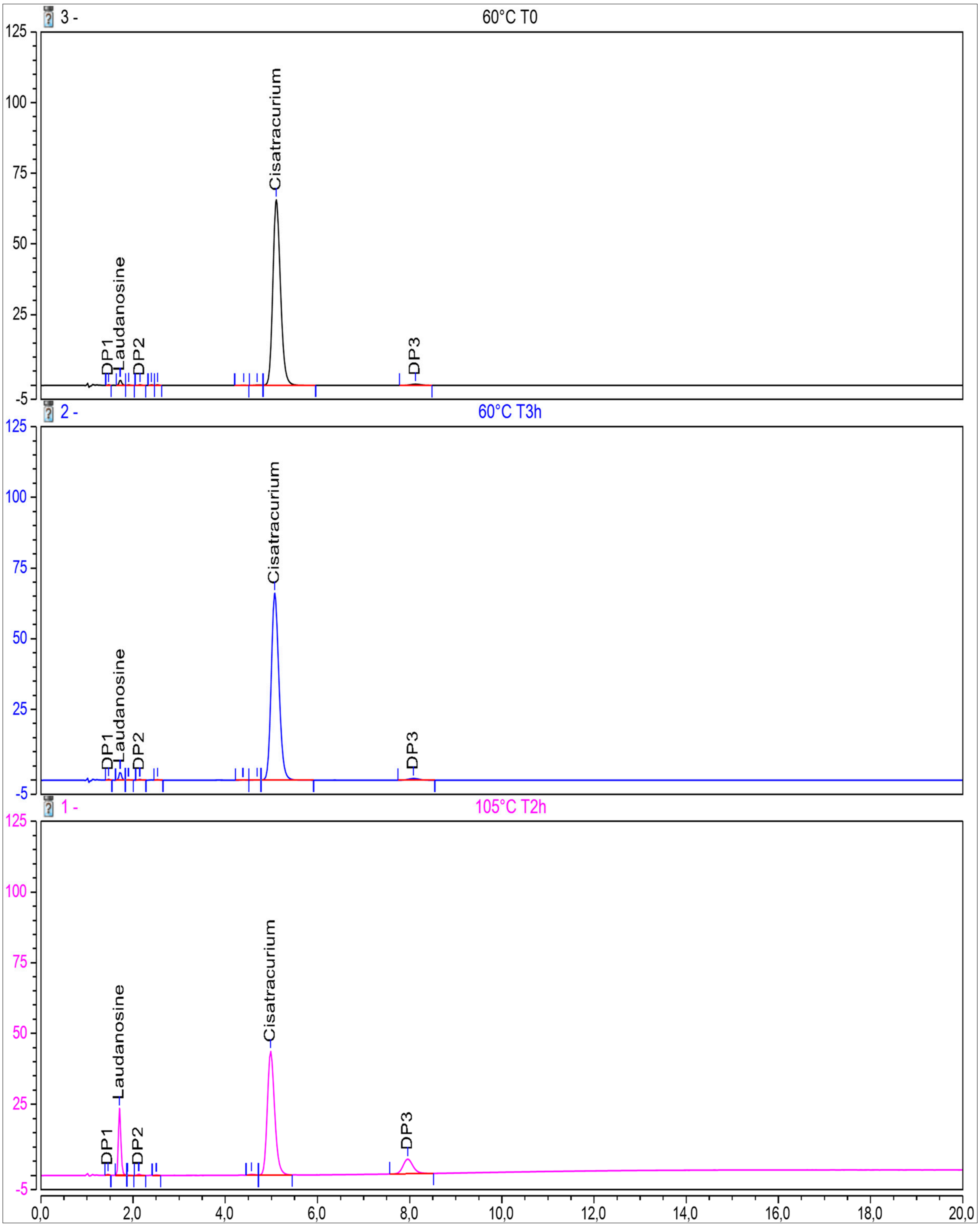

Figure S3. Chromatograms after the heat degradation of cisatracurium. (λ = 280nm).
T0: immediately after the start of heat exposure, T2h: After 2 hours of exposure at 105°C, T3h: After 3 hours of exposure at 60°.

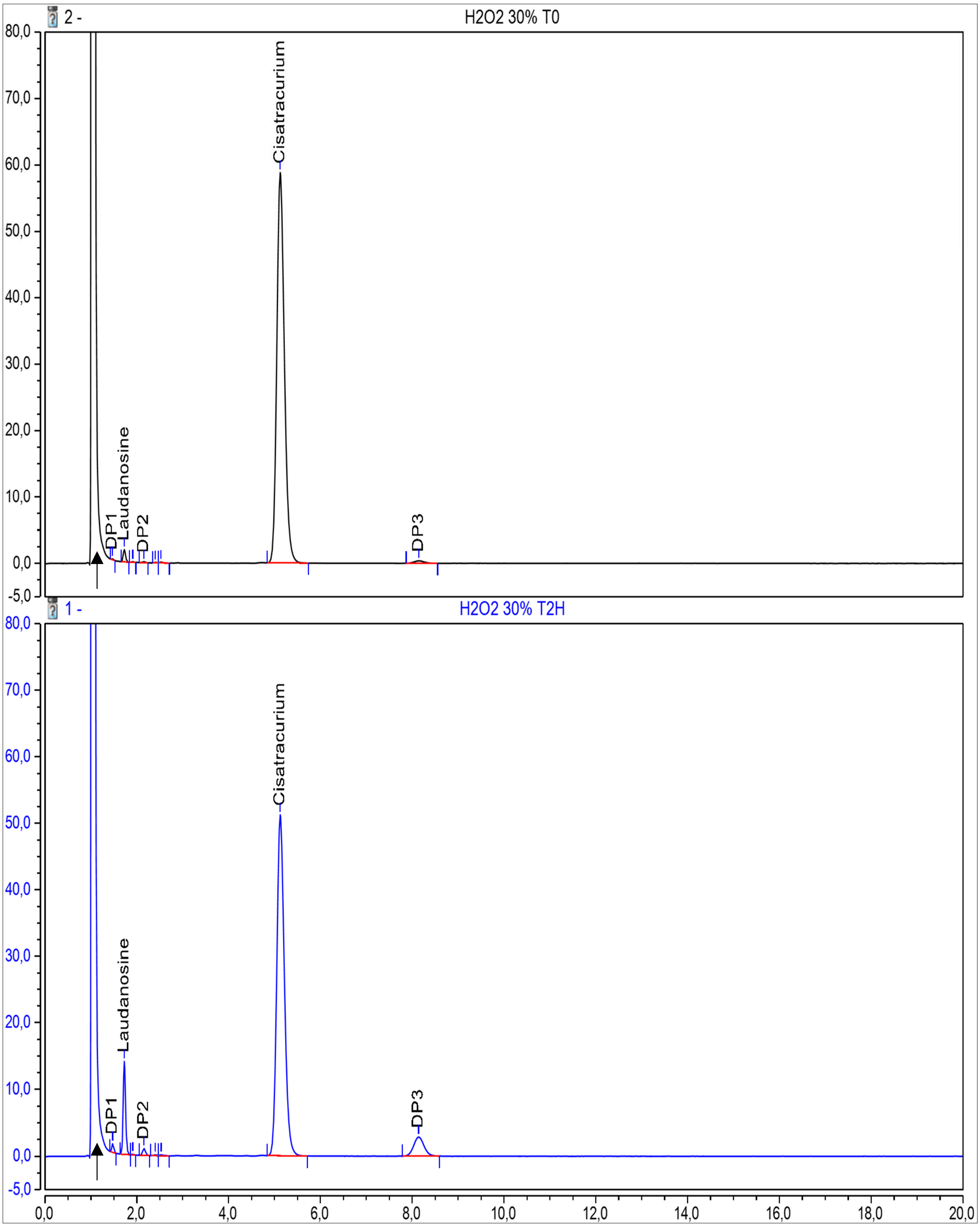

Figure S4. Chromatograms after degradation of cisatracurium by 30% H2O2 at 60°C. (λ = 280nm). The arrows indicate the peak corresponding to H2O2.

T0: immediately after the addition of H2O2; T2h: After 2 hours of exposure.

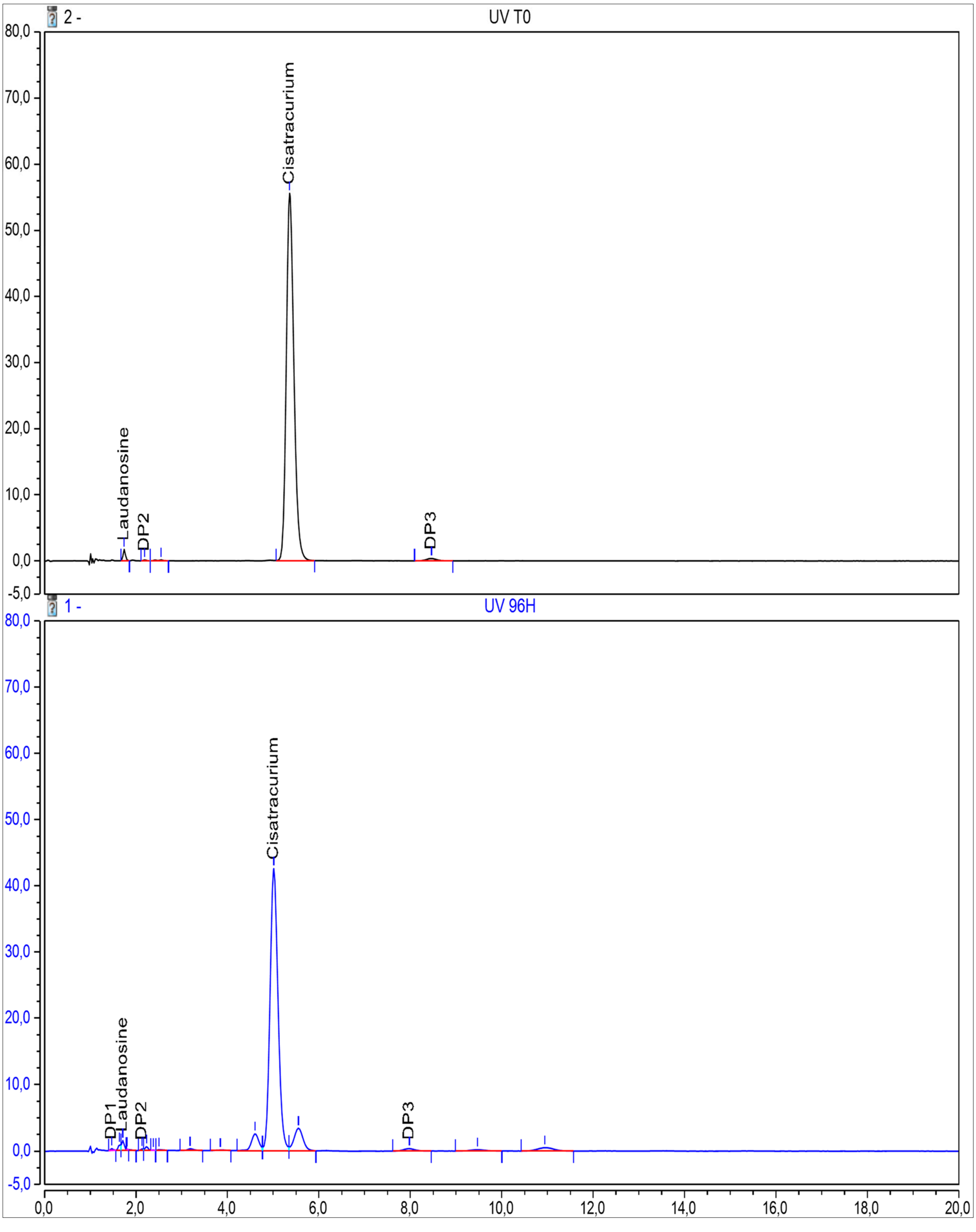

Figure S5. Chromatograms after UV degradation of cisatracurium at 254 nm. (λ = 280nm).

T0: immediately after the start of the UV exposure; 96h: after 96 hours of exposure.

Table S1. Stability study values at each time point - Data are given as mean ± standard deviation.

**Stability study time point**	**0**	**M3**	**M5**	**M7**	**M9**	**M12**	**M15**	**M16**	**M17**	**M18**
Cisatracurium (%C0)	100 ± 2.06	96.46 ± 2.34	95.62 ± 1.17	94.41 ± 3.07	93.99 ± 1.10	95.76 ± 1.85	94.21 ± 0.99	91.69 ± 1.25	89.77 ± 2.07	88.73 ± 2.35
Laudanosine (area%)*	0.21 ± 0.01	0.32 ± 0.02	0.37 ± 0.01	0.53 ± 0.02	0.75 ± 0.03	0.65 ± 0.01	0.76 ± 0.01	0.82 ± 0.01	0.90 ± 0.03	0.95 ± 0.03
DP1(area%)*	0.06 ± 0.00	0.47 ± 0.01	0.89 ± 0.02	1.16 ± 0.04	1.54 ± 0.02	2.02 ± 0.04	2.68 ± 0.03	2.99 ± 0.05	3.16 ± 0.06	3.30 ± 0.09
DP2 (area%)*	0.10 ± 0.00	0.52 ± 0.01	0.93 ± 0.02	1.20 ± 0.05	1.59 ± 0.02	2.05 ± 0.04	2.66 ± 0.03	2.95 ± 0.04	3.14 ± 0.06	3.29 ± 0.09
DP3 (area%)*	0.11 ± 0.01	0.20 ± 0.01	0.29 ± 0.01	0.35 ± 0.02	0.43 ± 0.01	0.50 ± 0.01	0.60 ± 0.02	0.65 ± 0.02	0.69 ± 0.01	0.71 ± 0.03
pH	3.43 ± 0.02	3.38 ± 0.08	3.24 ± 0.03	-	3.27 ± 0.01	3.22 ± 0.03	3.14 ± 0.01	3.05 ± 0.02	3.10 ± 0.04	3.10 ± 0.02
Osmolality (mOsm/kg)	25 ± 0	25 ± 0	26.33 ± 1.53	-	28.5 ± 0.71	23 ± 0.47	24.6 ± 0.84	24.5 ± 0.71	23.9 ± 0.32	24.7 ± 0.67
Particle size ≥ 10 µm (count/container)	200.70 ± 49.65	-	122.00 ± 12.59	-	201.67 ± 7.51	68.67 ± 19.70	-	-	-	119.33 ± 5.65
Particle size ≥ 25 µm (count/container)	3.60 ± 3.45	-	7.00 ± 2.35	-	2.00 ± 1.00	2.83 ± 1.47	-	-	-	4 ± 1.55
Endotoxin (IU/mL)	2.23 ± 0.40	-	-	-	-	4 ± 0	8 ± 0	-	-	8 ± 0

* Percentage peak areas for cisatracurium DPs are expressed as a percentage of the cisatracurium (base) peak area at the corresponding degradation time point

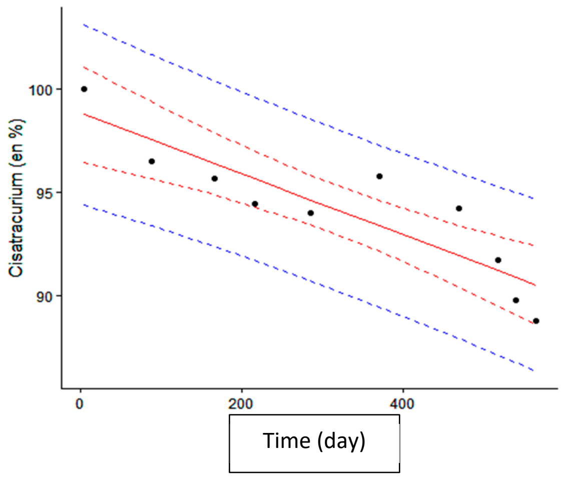

Figure S6. Linear fit in accordance with ICH Q1E.
The solid red line represents the linear fit applicable to our data. The dotted red line represents the limits of the calculated 95% confidence interval. The dotted blue line represents the 95% prediction interval.

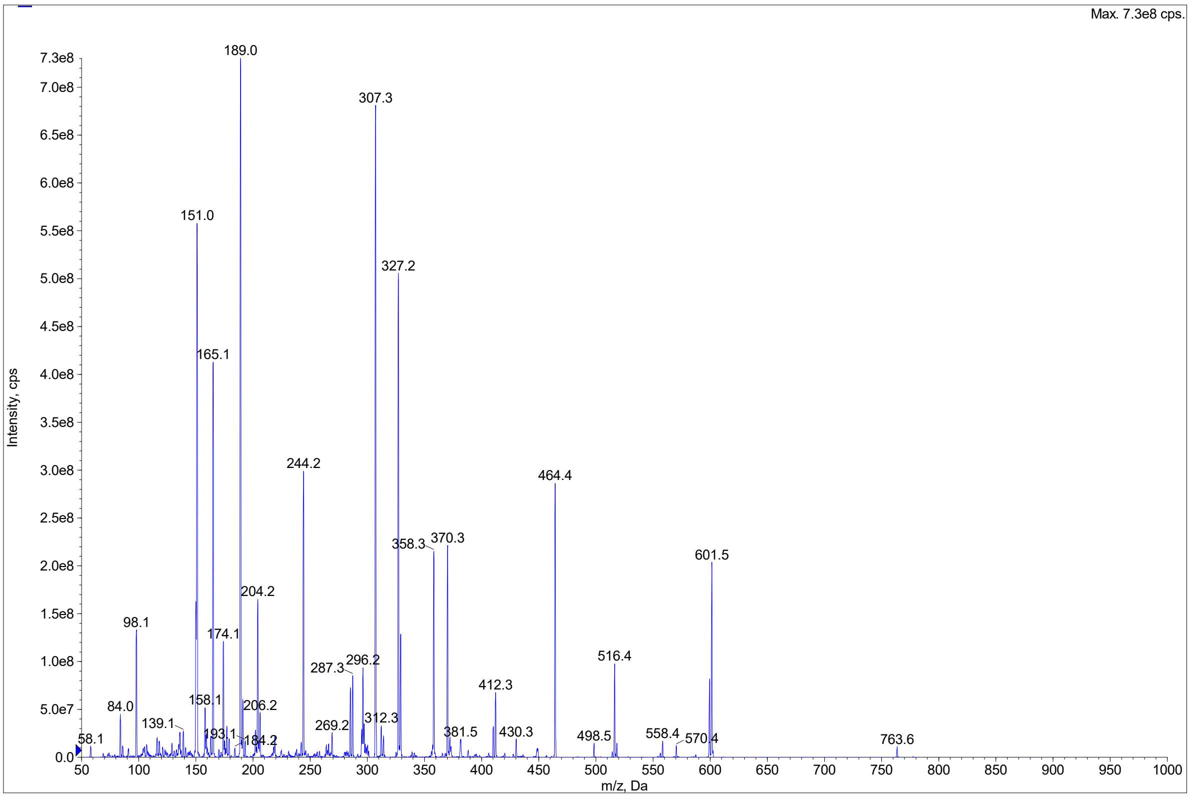

Figure S7. MS/MS spectrum of cisatracurium (m/z = 464 (M2+)), collision energy = 30 eV.

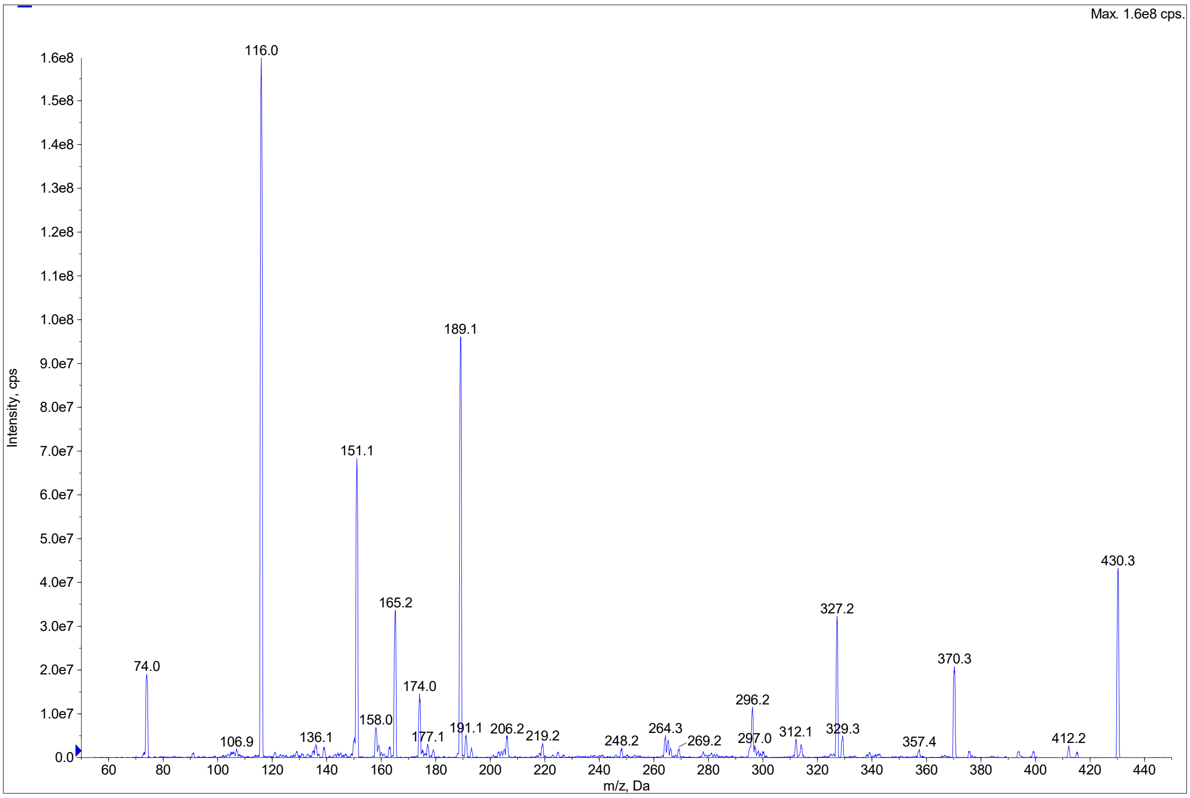

Figure S8. MS/MS spectrum of DP1, EP impurity A (m/z= 430 (M+)), collision energy = 35 eV.

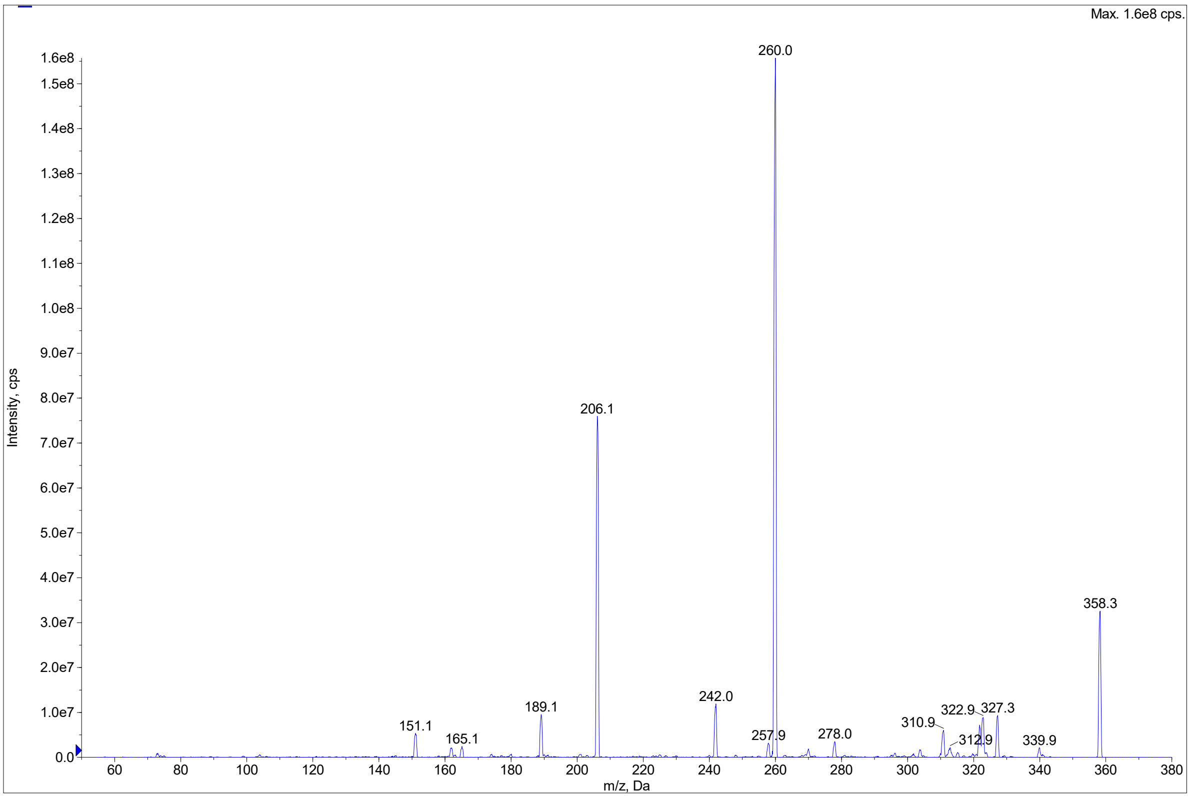

Figure S9. MS/MS spectrum of laudanosine, EP impurity C (m/z= 358 (M+H+)), collision energy = 25 eV.

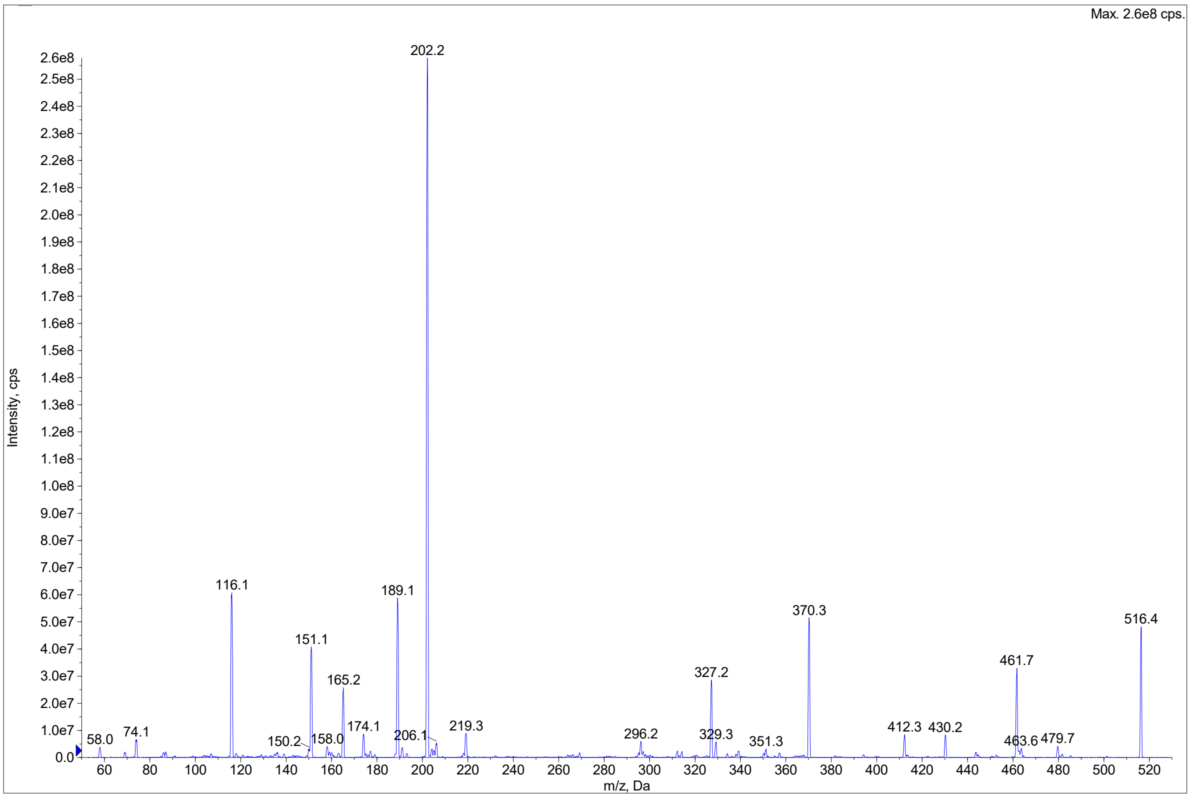

Figure S10. MS/MS spectrum of DP2, EP impurity E and/or F (m/z= 516 (M+)), collision energy = 40 eV.

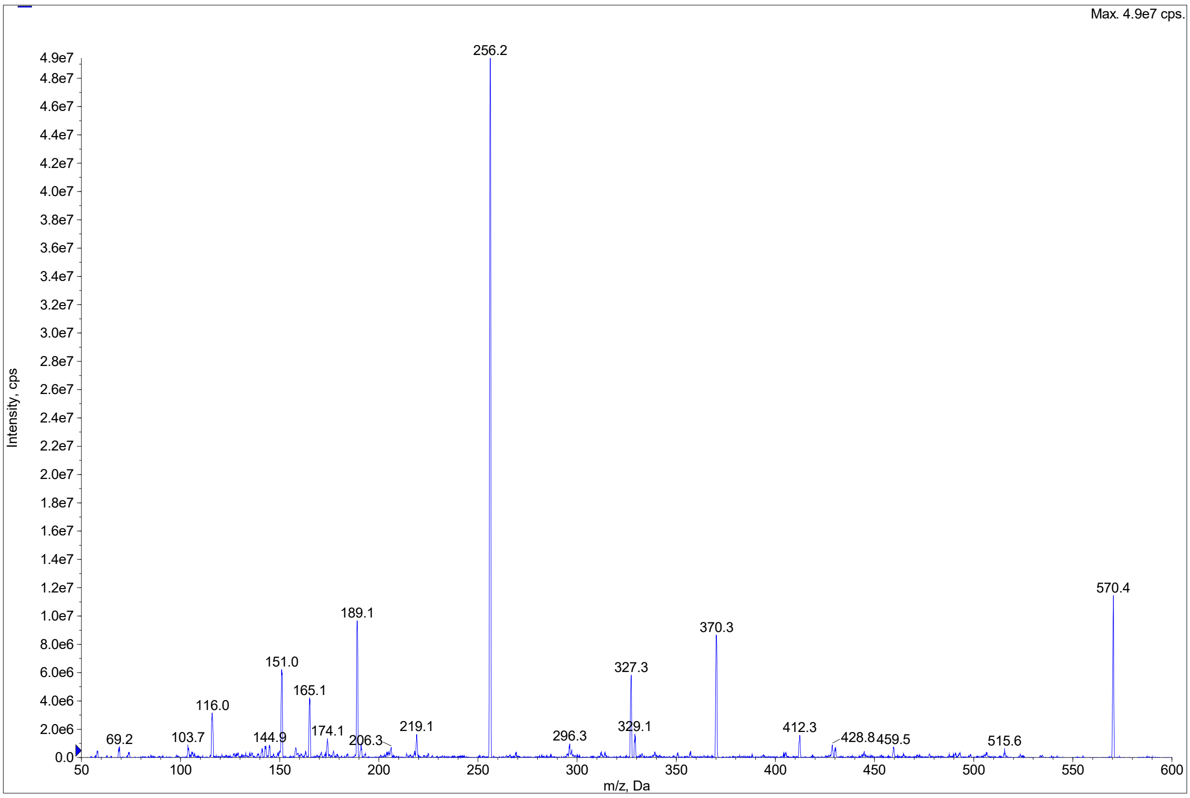

Figure S11. MS/MS spectrum of DP3, EP impurity N and/or O (m/z= 570 (M+)), collision energy = 40 eV.
